# DNA Methylation Biomarkers for Prediction of Response to Platinum-Based Chemotherapy: Where Do We Stand?

**DOI:** 10.3390/cancers14122918

**Published:** 2022-06-13

**Authors:** Nuno Tiago Tavares, Saulė Gumauskaitė, João Lobo, Carmen Jerónimo, Rui Henrique

**Affiliations:** 1 Cancer Biology & Epigenetics Group, Research Center of IPO Porto (CI-IPOP), RISE@CI-IPOP (Health Research Network), Portuguese Oncology Institute of Porto (IPO-Porto), Porto Comprehensive Cancer Centre (P.CCC), Rua Dr. António Bernardino de Almeida, 4200-072 Porto, Portugal; i12807@ipoporto.min-saude.pt (N.T.T.); i37297@ipoporto.min-saude.pt (S.G.); jpedro.lobo@ipoporto.min-saude.pt (J.L.); 2 Department of Pathology, Portuguese Oncology Institute of Porto, 4200-072 Porto, Portugal; 3 Department of Pathology and Molecular Immunology, School of Medicine & Biomedical Sciences, University of Porto (ICBAS-UP), Rua Jorge Viterbo Ferreira 228, 4050-513 Porto, Portugal

**Keywords:** cancer, platinum-based chemotherapy, epigenetics, DNA methylation, biomarker

## Abstract

**Simple Summary:**

Platinum-based agents are one of the most widely used chemotherapy drugs for various types of cancer. However, one of the main challenges in the application of platinum drugs is resistance, which is currently being widely investigated. Epigenetic DNA methylation-based biomarkers are promising to aid in the selection of patients, helping to foresee their platinum therapy response in advance. These biomarkers enable minimally invasive patient sample collection, short analysis, and good sensitivity. Hence, improved methodologies for the detection and quantification of DNA methylation biomarkers will facilitate their use in the choice of an optimal treatment strategy.

**Abstract:**

Platinum-based chemotherapy is routinely used for the treatment of several cancers. Despite all the advances made in cancer research regarding this therapy and its mechanisms of action, tumor resistance remains a major concern, limiting its effectiveness. DNA methylation-based biomarkers may assist in the selection of patients that may benefit (or not) from this type of treatment and provide new targets to circumvent platinum chemoresistance, namely, through demethylating agents. We performed a systematic search of studies on biomarkers that might be predictive of platinum-based chemotherapy resistance, including in vitro and in vivo pre-clinical models and clinical studies using patient samples. DNA methylation biomarkers predictive of response to platinum remain mostly unexplored but seem promising in assisting clinicians in the generation of more personalized follow-up and treatment strategies. Improved methodologies for their detection and quantification, including non-invasively in liquid biopsies, are additional attractive features that can bring these biomarkers into clinical practice, fostering precision medicine.

## 1. Introduction

Platinum-based agents (cisplatin (CDDP), carboplatin, and oxaliplatin) are broadly used for the treatment of several cancer types. Notwithstanding their broad spectrum of clinical use, several concerns remain, especially the emergence of treatment resistance, which causes additional challenges. Over the last decades, epigenetic biomarkers, especially those related to DNA methylation, have increasingly shown their value as cancer biomarkers, amenable for simple, fast, and low-cost detection in a non- or minimally invasive way. These are highly versatile, with value for diagnosis, risk stratification, and prediction of response to a specific treatment, sparing patients from harmful and unnecessary side effects. However, the setup and confirmation of a reliable biomarker with a strong routine clinical application is a complex process, which takes many steps from in vitro experiments to in vivo pre-clinical model validation and patient tissue analysis, with further validation in independent (multi-institutional) cohorts to ensure the desired high sensitivity, specificity, and accuracy [1,2]. Indeed, there is a plethora of studies proposing new biomarkers, but very few have made their way to clinical practice for several reasons including pre-analytical issues, cohort demographic variations, and a lack of standardized reporting, among many others [3]. In this review, we focused on epigenetic-based biomarkers, specifically DNA methylation, which might be used to predict response to platinum-based chemotherapy, emphasizing their establishment and detection methods.

### 1.1. Platinum-Based Chemotherapy: Brief Definition and Mechanisms of Action

Platinum anticancer drugs are routinely used for the treatment of several types of malignancies, both solid and hematolymphoid. Since the discovery of CDDP anti-tumor activity in 1969 and following its approval by the United States Food and Drug Administration (FDA) in 1978, platinum-based chemotherapy has been widely used in cancer treatment, which makes research on its mechanism of action and resistance extremely relevant for contemporary oncology [4,5]. Although CDDP remains the most commonly used platinum drug in the clinic, two analogs were also approved for tumor types: carboplatin (in 1989) and oxaliplatin (in 2002) [6,7].

Platinum-based chemotherapy has been proven to be effective to treat several types of cancers [8,9] and is widely used in the treatment of very distinct tumors, including esophageal (EC), gastric (GC), lung (LC) (small cell (SCLC) and non-small cell (NSCLC)), colorectal (CRC), and head and neck (HNC) cancers [10]. It is also used in urothelial (UC) and cervical (CC) carcinomas, as well as testicular and ovarian germ cell tumors (TGCT, OC). Platinum compounds are also used to treat other malignancies including leukemias, melanoma, neuroendocrine neoplasms, sarcomas, and tumors of neuroectodermal origin (such as neuroblastoma), demonstrating the versatility of these agents [11]. It is reasonable to theorize that such wide effectiveness in very distinct tumor types (with different biology, genomic drivers, risk factors, and molecular background) might be due to multiple pathways in which platinum-based drugs interfere.

Platinum compounds, especially CDDP, demonstrate remarkable clinical success in TGCT, enabling high cure rates (~80%) even in cases of heavily metastatic disease [12,13,14]. However, this specific type of testicular tumor shows hypersensitivity to CDDP, a fact that is tightly linked to its epigenetic and developmental biology background, and clinicians have not been able to reproduce such a success rate on somatic-type tumors of adulthood treated with the same platinum compounds [15,16,17]. This creates a need to fully understand the mechanism of action of this therapy and its mechanisms of resistance to improve patient care. In this review, we addressed the three platinum compounds more widely used in clinical practice, with a particular focus on the most widely used: CDDP.

CDDP has been used for years as a first-line treatment for several cancer types, either alone or in combination with other therapeutic options, such as radiation (to serve as a radiosensitizer) or other chemotherapeutics [18]. It is usually administered as either a neoadjuvant (for tumor shrinkage) or adjuvant (to lessen the risk of recurrence) therapy [19,20,21]. It may also be used in a palliative chemotherapy scenario, due to its cytotoxic activity, in an attempt to maintain patient quality of life [11]. The downside is that platinum agents demonstrate several critical side effects, such as nephrotoxicity and peripheral neurotoxicity, limiting the dose that might be used for patient treatment [22]. Additionally, cancer survivors previously treated with platinum disclose traceable levels of CDDP in urine and plasma many years after treatment, which is a major concern that may cause long-term side effects, triggering a decline in quality of life and, ultimately, resulting in death [23,24,25]. The current precision medicine paradigm is no longer compliant with sustaining such side effects either in a short and/or long term, and all efforts must be placed in improving risk stratification of patients with appropriate biomarkers to spare patients from futile, unnecessary treatments and their side effects.

Chemically, CDDP or cis-diamminedichloroplatinum (II) is formed by one platinum atom bound to two chloride atoms and two amide groups [26]. CDDP is known to cross the cell membrane by passive diffusion or through transmembrane transporters, of which the most studied are the copper transporters CTR1 and CTR2 [27,28]. The cytosol favors the aquation process of CDDP due to chloride concentration entailing CDDP activation [29]. Once inside the cell, CDDP binds strongly to the N7 reactive center of purine residues, causing DNA damage by creating adducts, blocking cell division, and resulting in apoptotic cell death [8,30]. CDDP, carboplatin, and oxaliplatin may cause different DNA adducts [30,31,32]; the fundamental cellular processes related to the CDDP mechanism are not fully understood and are subject to continuous study [33,34,35].

Carboplatin, also called cis-diamino-(1,1-cyclobutandicarboxylate) platinum (II), discloses a more favorable safety profile when compared to CDDP [36]. The downside is that carboplatin is much less potent, and, usually, a substantially higher clinical dose is required to match CDDP efficacy [37]. The mechanism of action of carboplatin is very similar to CDDP; previous works have demonstrated that CDDP-resistant tumor cell lines are cross-resistant to carboplatin as well [37].

Oxaliplatin has a 1,2-diaminocyclohexane carrier ligand [38]. Generally, oxaliplatin is more effective than CDDP in vitro [39]. However, single-agent oxaliplatin has low activity in many tumors clinically; thus, it is often combined with other drugs such as 5-fluorouracil (5-FU) [40,41]. Currently, it is mostly used in the treatment of advanced CRC [42]. Unlike CDDP and carboplatin, oxaliplatin reacts rapidly in plasma, undergoing a process of transformation into reactive compounds due to the displacement of the oxalate group [31].

### 1.2. A Brief Introduction to DNA Methylation

The radical difference between genetic and epigenetic changes is that genetic lesions are irreversible whereas epigenetic lesions are potentially reversible as they are associated with gain or loss of DNA methylation or other modifications of chromatin, thus enabling therapeutic intervention [43,44]. Epigenetic mutations, also called epimutations, are heritable; they may be constitutional and derived from a germline, thus expected to be found in all of the tissues of an individual, or they may be somatic, eventually restricted to a specific somatic tissue [45]. Epigenetic aberrations may consist of abnormal patterns of DNA methylation, disrupted patterns of histone posttranslational modifications (PTMs), altered expression of small non-coding RNAs, and alterations in chromatin composition and/or organization [46]. Histone modification and DNA methylation specifically regulate gene expression at the transcriptional level, preceding morphological changes associated with neoplastic transformation and even genetic alterations [47].

### 1.3. DNA Methylation Regulates Transcription and Affects Protein Levels

DNA methylation mostly affects CpG dinucleotides and is involved in tumorigenesis through three main mechanisms: locus-specific (e.g., tumor suppressor genes (TSG)) hypermethylation [48], global hypomethylation of the cancer genome [49], or direct mutagenesis of 5mC sequences [50,51]. It is noteworthy that all three routes occur simultaneously, indicating the importance of methylation as an epigenetic driver in cancer development. Hypermethylation negatively impacts transcription, reducing levels of the proteins responsible for processes such as DNA damage repair, creating a fundamental replication advantage over normal cells [52]. It was demonstrated that DNA methylation alterations result from the altered expression of methyltransferases [53]. Since these enzymes are responsible for the transfer of a methyl group to DNA, their up/downregulation leads to DNA hyper/hypomethylation, thus impairing normal epigenetic regulation and enabling malignant transformation and progression together with the increase in chemoresistance [54]. Importantly, due to their significant role in the epigenome regulation, methyltransferases could serve as a suitable therapeutic target in cancer treatment [55].

### 1.4. Epigenetic-Based Cancer Biomarkers

According to the National Cancer Institute (NCI) Dictionary of Cancer Terms, a cancer biomarker is a biological molecule that is found in blood or other body fluids or tissues, which indicates an abnormal, cancer-related process or condition [56]. Biomarkers vary depending on their objective (risk assessment, diagnosis, prognosis, or prediction of response to therapy), and herein we focus on the predictive type of biomarkers, which forecast the response to a specific treatment [57]. Ideally, a biomarker should have perfect (100%) specificity and sensitivity [57,58]. We reviewed available data on cancer cell lines and patient tissues because these are critical for the development of reliable biomarkers’ detection and establishment, including those based on the hypermethylation of gene promoters [59,60]. Recently, many studies focused on the need to identify and select reliable biomarkers for all kinds of cancer treatment. Sample (blood, urine, stool) collection is minimally or non-invasive and may thus be performed more frequently, allowing for easier and earlier diagnosis, disease monitoring, and easy storage [61]. Furthermore, it may assist clinicians in the decision of prescribing neoadjuvant or adjuvant treatment [59]. In this context, personalized medicine has become a priority. It is widely acknowledged that there is no universal treatment for cancer and that some patients are resistant to specific types of treatment. A perfect treatment strategy should target cancer cells in all the pathways that are crucial for their survival. Since epi-genetic aberrations may, at the least partially, contribute to cancer resistance to therapy and relapse, epigenetic modulation, such as DNA demethylating agents, may prove useful [62,63]. In this scenario, epigenetic-based biomarkers become relevant to determine whether a patient might benefit from a specific chemotherapy regimen, including those that are platinum based.

**Figure 1:**
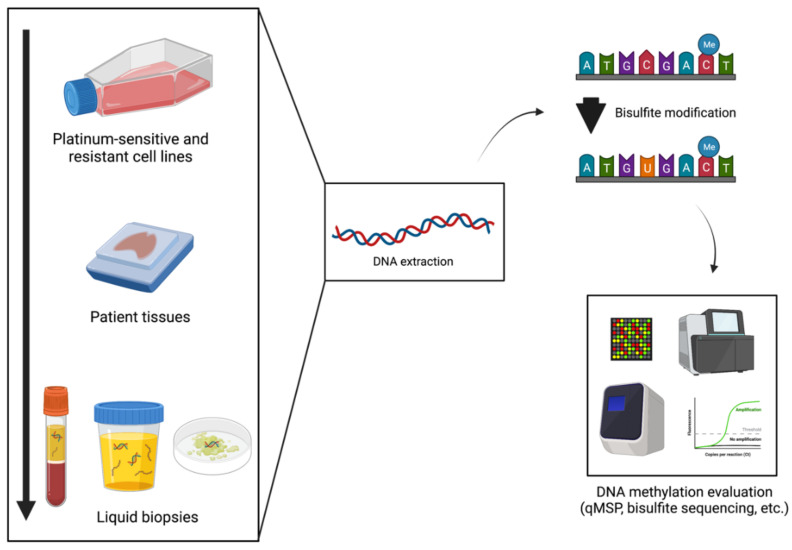
Platinum- resistance DNA methylation biomarkers’ examination process. Created with BioRender.com (accessed on 1 February 2022).

Among cancer-related epigenetic alterations, we focused our literature search on DNA methylation as a biomarker for predicting patient response to platinum-based chemotherapy (Figure 1). Indeed, DNA methylation itself is advantageous compared to other epigenetic biomarkers not only because it can be detected in non-invasively collected body fluids but also because it is representative of tumor heterogeneity. Whereas primary tumor and metastatic deposits’ tissue samples are highly heterogeneous, having several tumor cell clones, which may be missed by needle biopsy sampling, circulating tumor cells or nucleic acids are representative of the tumor bulk, either primary or metastatic [63,64]. Additionally, either in tissue or liquid biopsy specimens, DNA is much more stable and resistant to degradation (by formalin fixation, freeze, and thawing procedures) than RNA [57,65,66]. Furthermore, data obtained from the assessment of DNA methylation may be compared to absolute reference points (fully methylated/unmethylated DNA) allowing for quantification [65].

Currently, there is a large number of methods to detect DNA methylation, either target-based (e.g., methylation-specific polymerase chain reaction (PCR) (MSP), bisulfite sequencing, pyrosequencing methylation-specific restriction endonucleases, etc.) or genome wide-based (e.g., 450K or 850K array) and a vast amount of data are available publicly, enabling comparisons [67]. Finally, improvements in technology are under development to facilitate the detection of DNA methylation biomarkers in an absolute way without the need for pre-amplification reactions (e.g., droplet digital PCR (ddPCR)) [68,69].

Although such biomarkers seem auspicious due to their feasibility, they are not widely used in practice because of their limited sensitivity compared to the available standard-of-care tools. The particular reason for this circumstance is that often the detection of a single biomarker (e.g., promoter hypermethylation of a specific gene) is not sufficient to obtain a reliable conclusion and gene panels are required to overcome this limitation. Furthermore, for validation purposes, promoter methylation status must be confirmed using multiple methods and in several cohorts with distinct demographic features before. Additionally, other environmental conditions might impact gene methylation acting as confounders in cancer biomarker studies [67,70]. Additional problems are related to biomarker results’ interpretation and reporting, including normalization (appropriate normalizers, method of relative quantification), sample and DNA input conditions (which may be prohibitive in specific clinical scenarios), cost-related issues, etc.

To set up a reliable biomarker predictive of response to chemotherapy, one needs to identify relevant genes by compiling and testing training and validation cohorts and comparing DNA methylation status among specific cohorts of patients, specifically, in this setting, patients who responded (either completely or partially) to treatment and those who did not respond and endured a poor outcome [57,67,71]. Additionally, adjusting for demographic and clinicopathologic factors (age, gender, grade, stage, baseline characteristics of patients, etc.) is very relevant since DNA methylation biomarkers may lose their predictive value after adjustment in multivariable models. Moreover, cancer cell line testing is important to illuminate how chemotherapy-sensitive and -resistant cells react to treatment, evaluating whether the methylation profile changes over time and if the use of a demethylating agent sensitizes resistant cells [72]. For that purpose, the collection of patient tissue samples (for instance, biopsies, FFPE tissue samples, frozen samples, etc.), the extraction of DNA (assuring the best possible quality), and performing bisulfite treatment (or variations, such as with the use of methylation-sensitive endonucleases) followed by targeted MSP-based methods are required (Figure 1). Then, if sensitivity, specificity, positive predictive value (PPV), negative predictive value (NPV), and overall accuracy reach high levels of performance, a biomarker is a candidate for further testing in body fluids to determine whether it will constitute a reliable biomarker for clinical use [65].

## 2. Research Methodology

**Figure 2:**
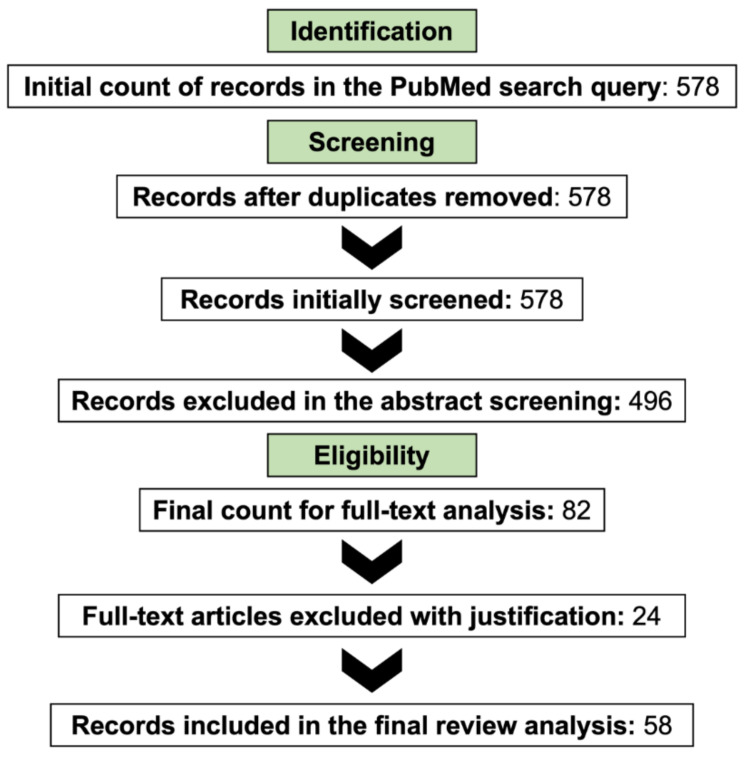
The research methodology employed for this review.

For the purposes of this review, a PubMed database search was conducted with the query (cisplatin OR carboplatin OR oxaliplatin) AND (DNA methylation OR epigenetics) AND (resistance OR chemoresistance). The search only considered original records published in English (i.e., reviews were excluded) and no restricted time interval was considered. Initially, the articles were chosen through comprehensive abstract analysis, and the final count was reached after a critical, full-text read of those that conveyed significant information for the topic. For this review, only studies analyzing the role of DNA methylation in platinum-based chemotherapy resistance using human cell lines or patient samples were considered. Figure 2 depicts the flow diagram representing the methodology used to reach the final set of selected sources of information. The information collected is summarized in Table 1 and Table 2, which depict epigenetically regulated genes associated with platinum-based chemotherapy resistance in cell lines (Table 1) or patient tissues (Table 2).

## 3. Discussion

### DNA Methylation and Platinum Resistance

Resistance to platinum treatment can be divided into two main types: intrinsic and acquired. Many patients that initially are sensitive to the treatment often develop resistance to it during their treatment course, causing relapse and reducing its overall clinical efficacy [29]. The development of new CDDP analogs, with fewer side effects, also aimed to tackle resistance to platinum-based chemotherapy, a goal which was not fully achieved and that also met with decreased effectiveness.

Changes in the epigenetic landscape, a cancer hallmark [129], appear to be nonrandom and are associated with the acquisition of chemoresistance to platinum in various types of cancers [130,131]. Indeed, platinum-based chemotherapy seems to induce changes in DNA methylation patterns [33,77,96,101,132]. This epigenetic mechanism plays a substantial role in the platinum resistance mechanism, affecting the transcription and translation of genes involved in reduced platinum influx to the cell or increased export (e.g., *ABCB1*), increased DNA damage repair routes (e.g., *BRCA1*, *ERCC1*, *MLH1*, *hMSH2*), inactivation of apoptosis pathways (e.g., *Casp8AP2*, *GULP1*, *p73*, *RIP3*), or increased platinum detoxification (e.g., *MT1E*) (Table 1 and Table 2). DNA damage repair pathways, for instance, have been proven to be of critical importance in the process of resistance to platinum compounds due to the DNA adducts these create [8,26,29,62,133]. The mismatch repair pathway (MMR) is a vital tool to keep genome stability, and a deficiency in this system has been shown to cause CDDP resistance in cells, associated with poor prognosis in some tumors [26,133]. Several previous studies have shown that promoter hypermethylation of genes involved in this pathway, such as *MLH1* and *hMSH2*, is associated with the acquisition of resistance to platinum therapy [72,92,105,110,121,124]. The NRF2/KEAP1 pathway plays a key role in the chemoresistance process of different tumor types and is capable of inhibiting apoptosis, promoting cell proliferation, and chemoresistance [134]. This pathway has already shown to be regulated by epigenetic modifications, including DNA methylation [135]. Additionally, genes such as *ERα* and ABC transporters, proven to be involved in the chemoresistance process, are regulated by the NRF2/KEAP1 pathway [136].

Our review of the literature disclosed several publications on the relevance of DNA methylation-based biomarkers for the prediction of response to platinum therapy, notwithstanding their heterogeneity concerning methodological settings (cell lines vs. tumor tissues). However, no such biomarker has been approved so far [57]. There are several critical steps in the validation of biomarkers as well as several hurdles that make the process of approval very strict, complex, time-consuming, and expensive, justifying why so very few of these markers make it all the way to clinical practice [2,57,65,71,137]. All these limitations make DNA methylation predictive biomarkers for platinum-based chemotherapy still relatively unexplored. One of the main problems observed in this type of study is the size of the validation cohort. If the cohort of patients treated with platinum compounds is not large enough, reliable conclusions about the predictive value of biomarkers cannot be drawn [74,92,99,105]. Thus, sample size estimation is mandatory to assure that the study cohort(s) enable the identification of significant differences between responders and non-responders if they exist. Additionally, multiple clinical variables must be considered, and the results should be adjusted/stratified according to these parameters, such as tumor type, stage, the platinum compound used for treatment, and treatment response, among others, as these are highly relevant clinical factors that may significantly influence methylation levels as well as the likelihood of response to therapy [57,71].

Another relevant issue is the estimation of tumor cell density in tissue samples tested. For example, the percentage of tumor in tissue sections chosen for DNA purification varies widely among published reports, from >30% of tumor cells [117] to >70% [82,125] or even >90% [98,122]. This variability is very likely to influence the determination of methylation levels (even considering that normalization for input has been made), jeopardizing the comparison of results and the reproducibility of experiments, undermining the possibility of biomarker validation [138].

An important and very common limitation in the publications assessed is the lack of information in clinical studies concerning biomarker performance parameters, such as sensitivity, specificity, accuracy, etc. [81,92,123]. These parameters are critical to evaluating the potential of the new DNA methylation biomarkers and comparing them with conventional methods or other markers that are routinely used in clinical care at present. Considering published data reviewed and depicted in Table 2, reported sensitivity or specificity values are modest (e.g., 67% [92] or 45% [81] sensitivity), probably owing to the very small size of initial tissue biopsies, which may not provide enough DNA for the experiments using several replicates, or contamination of purified DNA with tissue residues such as proteins, complicating the determination of correlation between gene methylation and expression [57,65,139,140]. To overcome this problem, optimal sample processing should be ensured and cohort size should be increased to account for variations in DNA concentration and purity among samples, enabling a more robust analysis of results [65].

Interestingly, differences in tissue condition regarding treatment are also apparent among the studies on methylation analysis. Whereas, in most studies, the tumor tissue analyzed for DNA methylation was collected after platinum treatment [108,109,117], in some assessed tumor tissue samples collected from untreated patients, primary cell cultures were established and were exposed to platinum prior to methylation analyses [88,98]. Results from these two strategies must be compared with caution because the presence or absence of the tumor microenvironment and altered cell communication derived from culture conditions is likely to entail the activation of different pathways [141].

Most clinical studies (i.e., those based on patient cohorts) performed DNA methylation analysis in tissues, either fresh, frozen, or formalin-fixed and embedded in paraffin. Indeed, very few have used liquid biopsies [75,103], which seem advantageous considering they are easier, less invasive, faster, and more comfortable to obtain compared with conventional tissue biopsies. Importantly, liquid biopsies allow for real-time monitoring, as blood or urine may be drawn periodically and biomarkers assessed over shorter or longer periods of time [137,142,143].

In addition to in vitro and clinical studies, in vivo animal models are very useful in cancer research as they more closely replicate the complexity and heterogeneity of cancer tissues compared to in vitro cell line studies [144]. Nonetheless, they are much more expensive and represent a superior work burden and some of these models may not very precisely mimic the human tumor microenvironment [145]. From our search, very few studies on DNA methylation biomarkers or therapeutic targets of platinum-based chemotherapy have used animal models and the ones that did mostly used those in in vivo assays to complement the in vitro cell studies [100,108,115].

Notwithstanding the hypothesis that DNA methylation biomarkers might help to predict a response to platinum-based chemotherapy, they may also represent important therapeutic targets that might help sensitize tumor cells to platinum compounds [72,74,80,86,95,99,107]. For instance, a previous study showed that the impairment of *ABCB1* expression due to promoter hypermethylation caused a reduction in the *ABCB1* transporter and lessened CDDP resistance [95]. Another study showed that promoter methylation levels of *BRCA1*, a key gene involved in DNA repair, were higher in CDDP-resistant ovarian cancer cell lines and that exposure to a demethylating agent sensitized those cells to platinum treatment [107]. Thus, if further demonstrated in clinical studies, DNA methylation patterns might allow for the improvement of therapeutic strategies [57,65,71].

**Figure 3:**
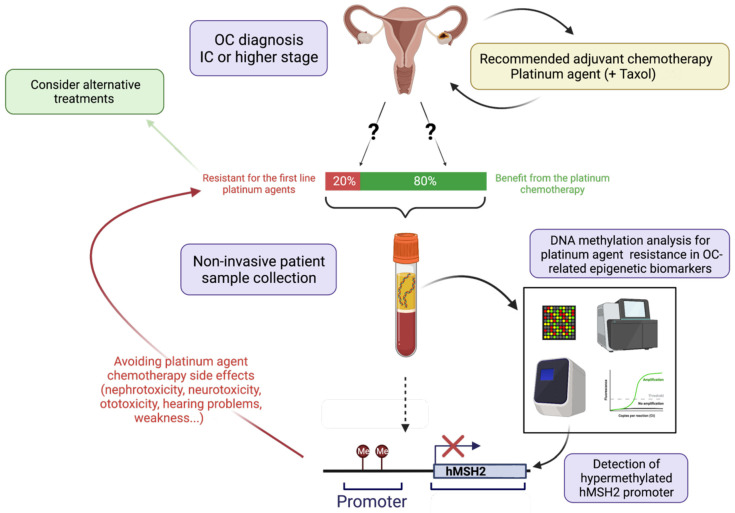
Schematic representation of pipeline for validation of DNA methylation-based biomarker to predict resistance to platinum-based chemotherapy in ovarian cancer (OC) patients. After a clinical diagnosis of OC (top of the picture), if the disease was staged as IC or higher, the recommended treatment is adjuvant chemotherapy with a platinum agent (CDDP, carboplatin, or oxaliplatin), eventually in combination with Taxol. However, there is a 20% probability that the patient will be resistant to platinum agents, which complicates the choice of treatment [66]. To select the best treatment method, biomarker validation could be performed. This follows with non-invasive patient sample collection (for instance, blood plasma), which can be used for circulating tumor DNA methylation analysis, focusing on platinum agent resistance. In this case, gene promoter hypermethylation indicating platinum resistance in OC was detected (e.g., *hMSH2*) [124], indicating that the patient will likely endure platinum resistance. Thus, not only may the side effects of ineffective treatment [22] be avoided but alternative treatments, eventually including epi-drugs, should be considered. Created with BioRender.com (accessed on 1 February 2022).

Figure 3 illustrates the ideal process of how a biomarker of platinum-based agent resistance (in this case, *hMSH2* promoter hypermethylation) could be validated and confirmed as a predictive biomarker, assisting in the therapeutic decision for OC patients, improving survival and quality of life.

Epi-drugs, which may be inhibitors of DNA methyltransferases, histone deacetylases, histone acetyltransferases, histone methyltransferases, or histone demethylases, may play an important role in cancer treatment by enhancing the effects of combinational therapy with platinum-based compounds as sensitizers [146,147]. This was shown in several clinical trials [73,148,149] and opens the way for a wider use of predictive DNA methylation-based biomarkers in tumors candidating for treatment with platinum compounds. Although holding substantial potential for the enactment of precision medicine, more robust validation studies are required to provide definitive evidence.

## 4. Conclusions

Presently, cancer is a leading cause of death worldwide and its incidence and mortality are increasing. Thus, in parallel with the implementation of preventive and early diagnosis measures, the development of effective and patient-specific therapeutic strategies is required to tackle this growing public health problem. Platinum-based chemotherapy, in use for more than 40 years, remains the first-line treatment for many types of cancer; resistance to this therapy is a major concern. Importantly, epigenetic dysregulation, specifically aberrant DNA methylation, plays an important role in the resistance process. Thus, biomarkers based on DNA methylation might enable the identification of those tumors more prone to demonstrate or acquire resistance to platinum compounds as well as constituting therapeutic targets enabling the sensitization of tumors. Thus, many studies have been undertaken to unveil and validate candidate biomarkers. Our review disclosed several mechanistic studies with cell lines and animal models, as well as some clinical studies, using patient samples, which identified some promising DNA methylation biomarkers predictive of response/resistance to platinum treatment. However, none of these biomarkers has been validated yet since most clinical studies analyzed small cohorts and the heterogeneity of patients, samples, and analytical methods precludes a meaningful and decisive conclusion. Hence, there is an urgent need to set up clinical validation studies, with adequate statistical power to enable the identification of the added value of those epigenetic biomarkers. This requires a joint effort from basic scientists and clinicians, departing from the more robust pre-clinical and clinical data available and bridging the gap that will lead to biomarker-assisted therapeutic decisions for patients who are candidates for platinum-based chemotherapy.

## Figures and Tables

**Table 1:** Promising DNA methylation markers predictive of resistance to platinum-based chemotherapy (mechanistic studies with cell lines). Genes related with the three most common platinum drug resistance pathways are indicated as [T], genes encoding proteins’ transporters, related with cellular uptake of platinum drugs; [R], genes encoding proteins responsible for DNA damage repair; or [A], genes encoding proteins related with the induction of apoptotic cell death.

Gene(s)	Platinum Compound	Tumor Model	Cell Line(s)	Methylation Detection Method	Key Findings	Ref.
*TLX3*	CDDP	BLCA	T24 and KK27	RLGS; COBRA; bisulfite sequencing	*TLX3* is involved in BLCA cell proliferation. *TLX3* gene promoter is hypermethylated in CDDP-resistant BLCA cell lines and hypomethylated in sensitive cells. *TLX3* methylation status in CDDP-resistant cells is 78.6%.	[73]
*HOXA9*	CDDP	BLCA (MIBC)	BC-3C, 647V, JO’N, BFTC-905, UM-UC14, RT4, 97-1, and 96-1	EpiTYPER™ assay	*HOXA9* promoter methylation status is related to response to CDDP-based chemotherapy in BLCA cell lines and metastatic BLCA. Demethylating agent-induced in vitro sensitization in resistant BLCA cell lines.	[74]
*GULP1*	CDDP	BLCA (UC)	SW780, UM-UC-3, BFTC909, RT4, 5637, BFTC905, HT1376, J82, T24, and HUC-1	MSP	CDDP-resistant T24 cell line discloses reduced endogenous *GULP1* expression. These cells have longer survival in response to CDDP, indicating a possible association between *GULP1* silencing and CDDP resistance.	[75]
*Casp8AP2* [A]	Oxaliplatin	CC	SiHa and S3	Differential methylation hybridization (DMH) microarray; qMSP; restriction with methylation-sensitive enzymes	There are global and individual loci methylation changes in resistant cells. Expression of *Casp8AP2* in oxaliplatin-resistant cells was reduced and associated with increased promoter methylation. After exposure to the demethylating agent, the sensitivity of resistant S3 cells was restored to the same level as in untreated SiHa cells.	[76]
*GPx3*	CDDP, oxaliplatin	CRC	RKO, SW48, LOVO, HCT116, SW480, SW620, COLO205, CACO2, and HT29	MSP	Cell lines tested showed different sensitivity to CDDP, and MSP analysis disclosed a correlation between *GPx3* methylation status and mRNA expression levels. Cell lines with *GPx3* promoter methylation and downregulation had increased sensitivity to platinum.	[77]
*PARPBP*	Oxaliplatin	CRC	HCT116 and HCT116L	Bisulfite sequencing	The methylation level of *PARPBP* promoter is decreased in the resistant cell line. A mechanism in resistant CRC cells was suggested, with *KIF18b* inhibiting the interaction between SP1 and *DNMT3b* through binding to *SP1*, resulting in hypomethylation of *PARPBP* promoter and consequent promotion of *PARPBP* expression. Then *PARPBP* promoted *PARP1* to enhance DNA repair in oxaliplatin-resistant cells.	[78]
*SLFN11*	CDDP	CRC	RKO, DLD1, SW620, LOVO, Ls180, and DKO	MSP; bisulfite sequencing	The expression of *SLFN11* is silenced by DNA methylation in CRC cell lines. *SLFN11* suppresses CRC cell proliferation and promotes chemosensitivity of CRC cells to CDDP in vitro.	[79]
*hMLH1* [R]	CDDP	EC	EC9706, EC1, EC9706-DDP, and EC1-DDP	MSP	*hMLH1* methylation in cell lines significantly increased after the acquisition of CDDP resistance. Resistance was reversed by exposure to a demethylating agent.	[72]
*CIDEA* [A]	CDDP	ESCC	KYSE30, KYSE140, KYSE150, KYSE180, KYSE410, KYSE510, and EC109	Bisulfite sequencing; MSP	KYSE410 cells with upregulated *CIDEA* disclose lower promoter methylation levels compared to KYSE30 and KYSE150 cells with downregulation of *CIDEA*. The 5-Aza-dC treatment restored the cellular expression of *CIDEA*. Gene downregulation was associated with promoter hypermethylation and the introduction of *CIDEA* enhanced sensitivity to CDDP.	[80]
*FGF5*	CDDP	ESCC	KYSE-30, 50, 140, 170, 180, 220, 270, 410, 450, 510, 520, and TE-15	Infinium^®^ HumanMethylation450K BeadChip; bisulfite sequencing	*FGF5* methylation is associated with response to chemoradiotherapy with CDDP. *FGF5* expression was induced by CDDP treatment in three unmethylated cell lines, but not in two methylated cell lines. Exogenous *FGF5* overexpression in a cell line with *FGF5* promoter methylation conferred resistance to CDDP.	[81]
*PAX5*	CDDP	ESCC	NUEC1 and TE3	qMSP	*PAX5*-silenced cells showed relatively higher cell proliferation and cell cycle promotion, suggesting acquisition of CDDP resistance due to methylation-associated gene silencing.	[82]
*BMP4*	CDDP	GC	AGS, Kato III, Hs746T, FU97, Ist1, MKN1, MKN7, MKN4, MKN28, MKN45, IM95, TMK1, AZ521, SCH, YCC3, YCC7, YCC10, YCC11, and YCC16	Bisulfite sequencing; MSP	Bisulfite sequencing of the *BMP4* 199 region confirmed that all five CpG sites within the region were fully methylated in CDDP-sensitive lines (YCC10, YCC11, YCC16, FU97) but unmethylated in CDDP-resistant cell lines (MKN45, AZ521, Kato III). *BMP4* was found methylated in sensitive, but not in resistant cells.	[83]
*CPT1C*, *KLK13*, *ETV7*, *FSCN1*, *NOTCH3*	CDDP	GC	Wild-type AGS and CDDP-resistant AGS	Infinium^®^ HumanMethylation450K BeadChip; bisulfite pyrosequencing	Expression of *KLK13*, *ETV7*, *FSCN1*, *CPT1C*, and *NOTCH3* before and after CDDP chemotherapy differed due to promoter methylation. These alterations may be associated with mechanisms of GC drug resistance and may be used as biomarkers to predict drug sensitivity.	[84]
*GTSE1*	CDDP	GC	AZ521, OCUM-1, SNU610, and SNU719	MSP	All hypomethylated cell lines depicted higher *GTSE1* expression. Loss of *GTSE1* expression significantly enhanced sensitivity to CDDP treatment as shown by a ~5-fold decrease in IC50 values in AZ521-kd cells. *GTSE1* knockdown in GC cells disclosed it as the major cause of CDDP resistance.	[85]
*SLFN11*	CDDP	GC	NUGC3, SNU5, SNU16, PHM82, NCI-N87, BGC823, MCG803, and AGS	MSP; bisulfite sequencing	*SLFN11* loss of expression was associated with promoter hypermethylation. Seven out of eight cell lines expressed *SLFN11* and the promoter region was methylated. *SLFN11* re-expression suppressed proliferation in SNU16 and MGC803 cell lines and sensitized cells to CDDP.	[86]
*CFLAR* [A], *ERBB2*, *KLF11*	CDDP	GCT	TCam-2	HumanMethylation27 DNA Analysis BeadChip (high-throughput methylation profiling)	Global methylation changes were determinant of the acquisition of resistance to CDDP, but methylation of some genes (*CFLAR*, *ERBB2*, *KLF11*) stood as the most promising markers to predict drug resistance.	[87]
*ERCC1* [R]	CDDP	Glioma	T98-G, UW28, MGR1, MGR2, and SF767	Genomic DNA methylation sequencing; MSP; real-time MSP	CDDP-sensitive MGR2 and SF767 cell lines disclosed methylation of *ERCC1* promoter CpG island (5.4 Kb upstream). CDDP inhibition rate was slightly reduced and CDDP killing efficiency was lower.	[88]
*CSF3R*	CDDP	HBL	HuH6 (wild-type and CDDP-resistant variant)	Infinium^®^ HumanMethylation450K BeadChip; bisulfite pyrosequencing	*CSF3R* was upregulated in CDDP-resistant cells after CDDP exposure compared to CDDP-sensitive cells. It was associated with methylation status.	[89]
*GPx3*	CDDP	HNC	PCI13, HN17B, HN22A, SCC25, SCC25cp, HN38, PCI51, FaDu, O11, and O12	Bisulfite DNA sequencing; MSP	Cell lines with *GPx3* promoter methylation depicted gene expression downregulation or total silencing. In *GPx3* methylated cells, 5aza-dC restored gene expression. CDDP-resistant and -sensitive cells significantly differed in *GPx3* promoter methylation levels. There was complete or partial *GPx3* methylation in 85% of CDDP-resistant HNC cells.	[90]
*NEFL*	CDDP	HNC	HaCaT, PCI13, O29, HN17B, HN22A, O12, HN38, O13, SCC25, O11, O22, PCI51, FaDu, SCC25cp, HN17Bcp, and O28	MSP	*NEFL* expression was observed in all CDDP-sensitive HNC cell lines and *NEFL* expression was absent or greatly reduced in all five cell lines displaying the highest level of CDDP resistance and in 2/5 of moderately resistant cell lines. The other 3 moderately sensitive lines showed high *NEFL* expression.	[91]
*CRIP1*, *G0S2*, *MLH1* [R], *OPN3*, *S100*, *TUBB2A*	CDDP	HNSCC	SCC-25 and SCC-25/CP	Methylight PCR	Methylation of these genes is associated with CDDP resistance. Decitabine treatment restored CDDP sensitivity in SCC-25/CP cells and significantly reduced the dose of CDDP required to induce apoptosis (sensitivity 67%, specificity 100%).	[92]
*MT1E*	CDDP	Melanoma	WM793, WM793-P1, WM793-P2, and 1205Lu	Bisulfite sequencing; MSP	*MT1E* promoter methylation is common in human melanoma and might be considered a biomarker. Gene silencing was suggested to play a role in the resistance of melanoma to chemotherapy.	[93]
*p73* [A]	CDDP, carboplatin	Multiple tumor types (CNS cancer, CRC, leukemia, melanoma, NSCLC, OC, PC, BC, RCC)	NCI-60 (panel of 58 cancer cell lines)	COBRA; MSP	A functional link between *p73* and alkylating agent (CDDP) sensitivity was confirmed, as in several cancer cell lines tested; downregulation of *p73* increased sensitivity to commonly used alkylating agents (CDDP and carboplatin).	[94]
*ABCB1* [T]	CDDP	LC	A549, A549/DDP	Bisulfite sequencing	*ABCB1* promoter methylation levels are significantly higher in CDDP-resistant cells compared to A549 cells.	[95]
*CLDN1*	CDDP	LC	CL1-0 and CL1-5	Bisulfite sequencing; MSP; pyrosequencing of CpG regions	*CLDN1* represses cancer progression via a feedback loop involving the *CLDN1*-*EPHB6*-*ERK1/2*-*SLUG* axis, which represses drug resistance and sensitizes lung adenocarcinoma cells to chemotherapy. DNA methylation maintains *CLDN1* expression. As *CLDN1* expression improves the efficacy of chemotherapy, it might constitute a biomarker predictive of response to chemotherapy.	[96]
*FOXF1*	CDDP	NSCLC	A549, A549/DDP, H1299, and 16HBE	Infinium^®^ HumanMethylation450K BeadChip; pyrosequencing	*FOXF1* promoter methylation levels are decreased in CDDP-resistant cells. *FOXF1* overexpression decreased CDDP-induced apoptosis of sensitive cells and *FOXF1* knockdown increased apoptosis of resistant cells.	[97]
*IGFBP-3*	CDDP	NSCLC	H23R, H460R, and 41R	Bisulfite sequencing	*IGFBP-3* is silenced by promoter hypermethylation in 41R and H23R-resistant cells compared with their parental sensitive cell lines, with marked *IGFBP-3* basal expression.	[98]
*RIP3*	CDDP	NSCLC	A549, H1568, H1299, H460, H23, H2009, H2023, H1689, HCC4006, Calu-3, and Calu-6	COBRA; Infinium^®^ HumanMethylation450K BeadChip	Hypermethylation of *RIP3* promoter region was detected in all LC cell lines but not in primary human bronchial epithelial cells. *RIP3* mRNA and protein expression increased after demethylating agent treatment in LC cell lines with methylated promoters, but not in those without methylation. Restored *RIP3* expression sensitized cells to CDDP.	[99]
*S100P*, *GDA*, *WISP2*, *LOXL1*, *TIMP4*, *ICAM1*, *CLMP*, *HSP8*, *GAS1*, *BMP2*	CDDP	NSCLC	A549 and A549/DDP	Infinium^®^ HumanMethylation450K BeadChip; qMSP	All candidate genes were hypermethylated in A549/DDP cells compared with parental A549 cells. In vivo studies also showed that *GAS1* downregulation by methylation was associated with CDDP resistance.	[100]
*SOX1*	CDDP	NSCLC	A549, A549/cis, H358, and H358/cis	Bisulfite genomic sequencing	*SOX1* is hypermethylated in CDDP-resistant cell lines compared to the parental cells. The expression of *SOX1* was upregulated in CDDP-resistant cells after treatment with demethylating agent. *SOX1* silencing enhanced CDDP-mediated autophagy in NSCLCs.	[101]
*TGM2*	CDDP	NSCLC	HCC-95, HCC-1588, NCI-H23, HCC-1195, NCI-H1299, HCC-2279, SK-MES-1, SK-LU-1, and HCC-1171	Bisulfite genomic sequencing	CDDP sensitivity was higher in *TGM2* promoter-methylated LC cell lines (HCC-95/1588) than in non-methylated ones (NCI-H1299 and HCC-1195). *TGM2* overexpression decreased sensitivity to CDDP and decreased *TGM2* expression, with siRNA in non-methylated cell lines increased sensitivity to CDDP.	[102]
*ECRG4*	CDDP	NPC	HNE1, HONE1, CNE1, SUNE1, CNE2, 6-10B, and C666-1	Bisulfite sequencing; MSP	Demethylation with 5-aza-dC induced reactivation of methylated and silenced *ECRG4* in NPC cell lines. NPC-derived cell line CNE1 was used for exogenous *ECRG4* overexpression, which increased tumor cell death when exposed to cisplatin.	[103]
*p57Kip2*	Carboplatin	OC (EOC)	PEO1, PEO1CisR, and PEO1CarbR	MSP; pyrosequencing	*p57Kip2* is epigenetically downregulated in a carboplatin-resistant cell line (PEO1CarbR). MSP analysis of the CpG island located at the 5’ region of the *p57Kip2* gene disclosed that methylation level was significantly higher for PEO1CarbR than for PEO1.	[104]
*ARHGDIB*, *PSMB9*, *HSPA1A*, *ARMCX2*, *MEST*, *FLNC*, *MLH1* [R], *MDK*, *GLUL*, *FLNA*, *NTS*, *COL1A1*, *NEFL*	CDDP	OC	A2780p5, A2780p6, A2780/cp70, A2780/MCP1, A2780/MCP6, PEO1, PEO4, PEO14, PEO23, PEA1, and PEA2	Array-based methylation profiling; pyrosequencing	Thirteen genes were consistently hypermethylated in CDDP-resistant A2780 cells; 5/13 genes (*ARMCX2*, *COL1A1*, *MDK*, *MEST*, and *MLH1*) acquired methylation in drug-resistant, OC-sustaining cells. *MLH1* gene was found to have a direct role in conferring CDDP sensitivity when reintroduced to cells in vitro.	[105]
*ASS1*	CDDP, carboplatin	OC	JAMA2, OVCA433, TR175, SKOV3, OVCAR3, 1847, A2780, and A2780 CisR	Bisulfite sequencing; MSP	There were methylated CpG dinucleotides in *ASS1* promoter of the CDDP-resistant A2780 CisR cell line whereas the parent A2780 cell line was not methylated. When *ASS1* was expressed in A2780 CisR and JAMA2 cell lines, the sensitivity to CDDP increased.	[106]
*BRCA1* [R]	CDDP	OC	COC1, COC1/DDP, and SKOV-3	qMSP; bisulfite genomic sequencing	CDDP-sensitive cells were found to harbor higher *BRCA1* promoter methylation levels than cells with inherent and acquired resistance. Treatment of cell lines with a demethylating agent decreased sensitivity to CDDP.	[107]
*FBXO32*	CDDP	OC	IOSE, HeyC2, SKOV3, MCP3, MCP2, A2780, and CP70	COBRA; MSP; real-time qMSP	*FBXO32* is downregulated in OC cells and its re-expression reduced tumor growth in vitro and in vivo. When restored in drug-resistant CP70 cells, *FBXO32* re-sensitized cells to CDDP and enhanced apoptosis, although, in more resistant HeyC2 cells, the re-expression only caused decreased cell cycle progression.	[108]
*FKBP1B*, *PAX9*	CDDP	OC	A2780 and OVCAR3 (and matched resistant variants)	Bisulfite sequencing; MSP; qMSP; whole-genome bisulfite sequencing; Infinium^®^ HumanMethylation450K BeadChip	*PAX9* and *FKBP1B* showed higher methylation levels in OVCAR3-resistant cell line compared to WT, control ovarian tissues, and PBMCs. There was a 4.7-fold increase in *FKBP1B* methylation comparing the resistant and sensitive variants of OVCAR3 and a 6-fold increase in *PAX9*. Moreover, *FKBP1B* overexpression caused increased CDDP sensitivity.	[109]
*hMLH1* [R]	CDDP	OC	A2780 parental cell line and 10 CDDP-resistant A2780 derivative cell lines	Promoter DNA restriction with methylation-sensitive (HpaII) and methylation-insensitive (MspI) endonucleases; Southern blot analysis	*hMLH1* promoter methylation was confirmed and loss of protein expression was observed. The CDDP-sensitive parental cell line was methylated only in one of the *hMLH1* promoter alleles, whereas the resistant one was methylated in both alleles.	[110]
*NAGA*	CDDP	OC, NSCLC	PA-1, TOV-21G, TOV-112D, Caov-3, A2780, A2780cis, MDAH2774, ES-2, OVCAR-3, OV-90, and SK-OV-3	Infinium^®^ HumanMethylation450K BeadChip	*NAGA* mRNA downregulation correlated with specific *NAGA* promoter CpG site hypermethylation in CDDP-resistant OC cells. Demethylating agent restored expression and CDDP cytotoxicity increased, whereas loss of *NAGA* induced increased chemoresistance in sensitive and resistant cells.	[111]
*OXCT1*	CDDP	OC	SK-OV-3, PA-1, Caov-3, TOV-21G, A2780, TOV-112D, OV-90, and OVCAR-3	Infinium^®^ HumanMethylation450K BeadChip	*OXCT1* downregulation by hypermethylation of CGI within the promoter region is significantly higher in CDDP-resistant cell lines than in the sensitive ones. In the most resistant SKOV3 OC cell line, *OXCT1* overexpression improved sensitivity to CDDP.	[112]
*SFRP5*	CDDP	OC	SKOV3, A2780s, CP70, and OVCAR3	MSP; bisulfite sequencing	All tested OC cell lines disclosed *SFRP5* hypermethylation. Treatment with methylation inhibitor restored *SFRP5* mRNA expression. Epigenetic silencing of *SFRP5* affected tumor growth, invasion, tumorigenicity, and chemosensitivity of OC cells.	[113]
*SLFN11*	CDDP, carboplatin	OC, NSCLC	SK-OV-3 and NCI-H23	Bisulfite sequencing; Infinium^®^ HumanMethylation450K BeadChip	When *SLFN11* was downregulated by shRNA, both cell lines showed significantly increased IC50 values for platinum treatment compared to control cells, indicating a role for *SLFN11* in platinum resistance.	[114]
*TMEM88*	Carboplatin	OC	A2780 (injected in mice)	Infinium^®^ HumanMethylation450K BeadChip	In mice injected with A2780 cells and treated with carboplatin hypomethylation of *TMEM88*, gene promoter in resistant tumors was observed. It was confirmed that *TMEM88* mRNA expression levels are increased in resistant tumors versus controls, which is consistent with gene promoter hypomethylation in those tumors.	[115]
*TRIB2*	CDDP	OC	A2780, SKOV3, and HeyA8	Microarray-based methylation analysis	Analysis of *TRIB2* confirmed an indirect contribution of hypermethylation to gene silencing and the functional impact of this gene on A2780 chemosensitivity. *TRIB2* overexpression in resistant cells led to reduced IC50, and shRNA-mediated silencing of *TRIB2* in parental sensitive A2780 cells increased their resistance.	[116]

**Table 2:** Promising DNA methylation markers predictive of resistance to platinum-based chemotherapy (studies with patient samples). Genes related with the three most common platinum drug resistance pathways are indicated as [T], genes encoding proteins’ transporters, related with cellular uptake of platinum drugs; [R], genes encoding proteins responsible for DNA damage repair; or [A], genes encoding proteins related with induction of apoptotic cell death.

Gene(s)	Platinum Compound	Tumor Model	Sample Type	Sample Grouping and Size	Patients’ Gender and Mean Age	Methylation Detection Method	Key Findings	Ref.
*ERα*	CDDP	BC (TNBC)	Tumor tissue samples	35 patient samples	All ♀ (median age 47 y.o., range 27–69 y.o.)	MSP	Tumor samples with *ERα* methylation were resistant to CDDP. Furthermore, *ERα* methylation was related to increased *BRCA1* expression, indicating a possible resistance mechanism.	[117]
*TLX3*	CDDP	BLCA	Tumor tissue samples	110 patient samples	n.m.	RLGS; COBRA; bisulfite sequencing	*TLX3* is hypermethylated in tumors resistant to CDDP. Methylation in patient samples and cell lines was congruent, indicating a role for *TLX3* as a biomarker of response to CDDP.	[73]
*HOXA9*	CDDP	BLCA (MIBC)	Tumor tissue samples from vesical transurethral resections	18 patient samples	15 ♂ and 3 ♀; mean age 69 y.o. at the time of cystectomy (median 71, range 60 to 77 y.o.)	EpiTYPER™ assay	*HOXA9* promoter methylation status was associated with response to CDDP-based chemotherapy in MIBC. *HOXA9* promoter methylation might be used to predict sensitivity or resistance to CDDP-based chemotherapy in BLCA patients.	[74]
*GULP1*	CDDP	BLCA (UC)	Tumor tissue and urine samples	46 urine samples from individuals without neoplastic disease; 58 diagnosed with UCB; 20 primary tumors and matched normal samples; 76 primary tumors	n.m.	MSP	The qMSP in tumor samples showed a significantly higher frequency of *GULP1* promoter methylation in tumors than in matched normal tissues. The results were confirmed in urine samples and TCGA-BLCA dataset. *GULP1* might be a biomarker of resistance to CDDP.	[75]
*p73* [A]	CDDP	BLCA (MIBC)	Tumor tissue samples	14 patient samples (8 low and 6 high methylation)	n.m.	Infinium^®^ HumanMethylation450K BeadChip; pyrosequencing	The *p73* promoter methylation was significantly related to worse OS (high methylation: 13.5 months vs. low methylation: 30 months). The *p73* promoter hypermethylation might be a predictive biomarker for CDDP response in BLCA patients.	[118]
*SLFN11*	CDDP	CRC	Tumor tissue samples	133 patient samples (128 primary CRC cases and 5 noncancerous colorectal mucosae)	84 ♂ and 44 ♀ (30 < 50 y.o. and 98 ≥ 50 y.o.)	MSP; bisulfite sequencing	*SLFN11* was found methylated in 55.47% of human CRC samples, regulating gene expression. *SLFN11* methylation is significantly associated with age, poor 5-year OS, and RFS.	[79]
*TFAP2E*	Oxaliplatin	CRC	Tumor tissue samples	74 patient samples (metastatic CRC)	n.m.	MethylLight	The cohort treated with oxaliplatin disclosed a negative association between methylation and treatment response: higher response rates among patients with hypomethylated *TFAP2E* (3/20 patients with hypermethylated *TFAP2E* responded to treatment, whereas 33/54 patients with hypomethylated *TFAP2E* responded).	[119]
*FGF5*	CDDP	ESCC	Tumor tissue samples	117 patient tumor samples of responders and non-responders (41 patients in screening set, 44 patients in validation set, 42 patients in re-validation set)	Screening set: 34 ♂, 7 ♀ (mean age 64.6 y.o.); validation set: 28 ♂, 6 ♀ (mean age 66.8 y.o.); re-validation set: 30 ♂, 9 ♀ (mean age 65.9 y.o.)	Infinium^®^ HumanMethylation450K BeadChip; bisulfite sequencing	*FGF5* methylation might be a biomarker predictive of sensitivity to dCRT (with CDDP). Methylome screening identified the specificity of *FGF5* expression and associated promoter methylation levels with the response (45% sensitivity and 90% specificity in the combined validation and re-validation sets, n = 76).	[81]
*PAX5*	CDDP	ESCC	Tumor tissue surgical samples	156 ESCC patient samples (78 tumor and 78 normal adjacent)	62 ♂ and 16 ♀, 37 ≥ 65 y.o. and 41 < 65 y.o.	qMSP	*PAX5* methylation was frequent and highly tumor specific in ESCC. Methylation was significantly associated with low protein expression in tumors. *PAX5* silencing correlated with increased cancer cell proliferation and CDDP resistance and might associate with poor RFS.	[82]
*BMP4*	CDDP	GC	Tumor tissue samples	197 patient samples	n.m.	Bisulfite sequencing; MSP	A significant correlation between *BMP4* methylation status and mRNA expression was found across tumors investigated. *BMP4*- expressing tumors were associated with poor GC prognosis and possible resistance to CDDP.	[83]
*MLH1* [R]	Oxaliplatin	GC	FFPE tumor tissue samples	53 oxaliplatin-treated patient samples	72 ♂ and 30 ♀, median age 53 y.o.	Nested MSP	In oxaliplatin-treated patients, *MLH1* methylation was found in 30.2% of cases. OS was higher in the unmethylated *MLH1* group vs. methylated group (*p* = 0.046). Patients with methylated *MLH1* promoters were found to be resistant to oxaliplatin. *MLH1* methylation might be an oxaliplatin-resistance biomarker in GC.	[120]
*SLFN11*	CDDP	GC	Tumor tissue samples	209 patient samples (201 GC samples and 8 normal gastric mucosa samples)	157 ♂ and 44 ♀ (39 patients < 50 y.o. and 162 patients ≥ 50 y.o.)	MSP; bisulfite sequencing	*SLFN11* was found methylated in 29.9% of human GC samples, and *SLFN11* expression was regulated by promoter methylation. Additionally, *SLFN11* methylation was significantly associated with tumor size.	[86]
*ERCC1* [R]	CDDP	Glioma	Tumor tissue surgical samples	32 patient samples	1 8♂ and 14 ♀ (median age 29 y.o.)	MSP; real-time MSP	Aberrant *ERCC1* promoter methylation was found in primary glioma samples. *ERCC1* mRNA and protein expression levels, as well as response to CDDP in glioma, were associated with *ERCC1* promoter methylation levels.	[88]
*APC*, *RASSF1A* [A], *HIC1*, *BRCA1* [R], *MGMT*, *RARB*, *FHIT*, *FANCF* [R], *ECAD*	CDDP	Male GCT	Tumor tissue samples	70 patient samples (31 CDDP-sensitive and 39 resistant)	n.m.	MSP	One or more genes were methylated in 59% of tested tumors. The top hypermethylated genes were *RASSF1A* (35.7%), *HIC1* (31.9%), *BRCA1* (26.1%), and *APC* (24.3%). *RASSF1A* and *HIC1* inactivation by promoter hypermethylation might constitute biomarkers for platinum resistance.	[121]
*CSF3R*	CDDP	HBL	Fresh-frozen tumor samples	43 patient samples (38 CDDP-sensitive and 5 resistant)	n.m.	Infinium^®^ HumanMethylation450K BeadChip; bisulfite pyrosequencing	*CSF3R* hypermethylation was evaluated in CDDP-resistant hepatoblastoma. *CSF3R* hypermethylation was associated with CDDP resistance and might assist in selecting ion of HBL patients for postoperative chemotherapy.	[89]
*GPx3*	CDDP	HNC	Frozen tumor tissue samples	46 patient samples	34 ♂ and 12 ♀; mean age: 43.8 ± 24.3 y.o.	Bisulfite sequencing; qMSP; MSP	61% of tested HNC primary tumors harbored *GPx3* methylation vs. only 8% of normal tissue samples. In cases with complete or partial response to chemotherapy, 82.6% of samples were not methylated and 59% of patients with no response to chemotherapy disclosed methylated *GPx3*.	[90]
*NEFL*	CDDP	HNC	Frozen tumor tissues	51 patient samples (25 methylated and 26 unmethylated for NEFL)	39 ♂ and 12 ♀	Bisulfite DNA sequencing; MSP; real-time MSP	Patients with methylated *NEFL* promoter were nearly 3 times more likely to endure resistance to CDDP-based chemotherapy. *NEFL* methylation also predicted reduced OS and disease-free survival in HNC patients who received CDDP-based chemotherapy.	[91]
*CRIP1*, *G0S2* [A], *MLH1* [R], *OPN3*, *S100*, *TUBB2A*	CDDP	HNSCC	FFPE tumor samples	19 patient samples (10 progressed, 2 stable, and 7 complete remission 6 months post-CDDP treatment)	12 ♂ and 7 ♀; mean age 57.95	Methylight PCR	The genes tested disclosed higher promoter methylation in CDDP-resistant than CDDP-sensitive tumors. Genes were assembled into a classifier, which might be used to categorize sensitivity to CDDP (67% sensitivity, 100% specificity).	[92]
*GDA*, *S100P*, *WISP2*, *LOXL1*, *TIMP4*, *ICAM1*, *HSP8*, *GAS1*	CDDP	NSCLC	Primary tumor samples	40 patient samples (20 CDDP-resistant and 20-sensitive)	n.m.	Infinium^®^ HumanMethylation450K BeadChip platform; qMSP	The genes listed were found to disclose higher methylation levels in CDDP-resistant NSCLC samples compared to sensitive tumors.	[100]
*IGFBP-3*	CDDP	NSCLC	Paraffin-embedded surgical specimens	36 patient samples (19 CDDP-resistant and 17 sensitive); 10 control biopsies	34 ♂ and 2 ♀; mean age 65.8 y.o.	Bisulfite sequencing; MSP	Most CpG dinucleotides were methylated in resistant but not in sensitive primary tumors, indicating a significant association between *IGFBP-3* methylation and CDDP chemosensitivity.	[98]
*IGFBP-3*	CDDP	NSCLC	Paraffin-embedded surgical specimens	25 patient samples	23 ♂ and 2 ♀; mean age 63.7 y.o.	MSP	*IGFBP-3* promoter methylation and IGFIR/AKT phosphorylation occurred only in CDDP-resistant NSCLC patients. *IGFBP-3* deficiency due to methylation might mediate the resistance to CDDP through activation of IGFIR/AKT pathway.	[122]
*LRP12*	Carboplatin	NSCLC	FFPE primary tumor samples and frozen tumor tissue samples	PDX models derived from 22 NSCLC patients and validation in an independent cohort of 35 patient FFPE samples	n.m.	Me-DIP Seq; targeted bisulfite sequencing; MSP	*LRP12* hypermethylation correlated with increased resistance to carboplatin. *LRP12* methylation was significantly higher in patients with relapse (13.9% vs. 7.4%). A threshold of 8.3% was determined, allowing us to classify tumors into responders and non-responders to carboplatin (80% sensitivity, 84% specificity).	[123]
*RIP3*	CDDP	NSCLC	Frozen tumor tissue samples	16 NSCLC patients (both normal and tumor tissues)	n.m.	COBRA; Infinium^®^ HumanMethylation450K BeadChip	The quantitative methylation data for probes located within the *RIP3* promoter CpG island revealed significantly higher methylation in 25% of tumors. When *RIP3* promoter was methylated, protein expression was suppressed, correlating with increased resistance to CDDP.	[99]
*hMSH2* [R]	CDDP, carboplatin	OC (EOC)	Patient tissues	40 patient samples (18 CDDP-resistant and 22-sensitive)	Median age 56 y.o. (years old)	RRBS; MALDI-TOF mass spectrometry	A specific promoter region containing CpGs was significantly hypermethylated in platinum-resistant patients. High *hMSH2* promoter methylation levels are associated with poor prognosis in patients submitted to CDDP treatment.	[124]
*DLG2*, *OR51L1*, *OR51I1*, *OR51F1*, *OR51B6*, *HBBP1*, *TMEM200A* [T]	CDDP	OC (HGSOC)	Frozen cryosections of tumor tissue samples	30 patient samples	10 platinum-sensitive cases (60% did not recur in 5 years); 20 platinum-resistant cases (5% did not recur in 5 years after treatment)	Illumina 850K methylation assay	The platinum-sensitive group depicted lower methylation levels than the platinum-resistant group. In an epigenome-wide association study, differentially methylated probes helped to identify hypermethylated genes in platinum-resistant patients.	[125]
*EGR1*, *MGRN1*	CDDP	OC (HGSOC)	Tumor tissue surgical samples	96 patient samples (55 platinum-sensitive and 41 platinum-resistant)	34 < 50 y.o. and 62 ≥ 50 y.o.	RRBS; MALDI-TOF mass spectrometry	The promoters of *MGRN1*, *EGR1* were significantly hypermethylated in cancer tissues from platinum-resistant HGSOC. Lower *MGRN1* and *EGR1* expressions due to hypermethylation were associated with clinical outcomes.	[126]
*FZD10*	CDDP	OC (HGSOC)	Frozen tumor tissue samples	70 patient samples divided by 2 patient groups (group 1: 18 advanced-stage HGSOC samples; group 2: 21 responder and 31 non-responder samples)	Group 1: median age 61 y.o.; group 2: median age 62.25 y.o.	MethylCap-seq; bisulfite pyrosequencing	*FZD10* was the most differentially methylated gene among two of the chemoresponsive-related groups. *FZD10* expression was significantly lower due to promoter methylation in the extreme responder HGSOC patient group compared to the non-responder group.	[127]
*ARHGDIB*, *PSMB9*, *HSPA1A*, *ARMCX2*, *MEST*, *FLNC*, *MLH1* [R], *MDK*, *GLUL*, *FLNA*, *NTS*, *COL1A1*, *NEFL*	CDDP	OC	Tumor tissue samples	14 patient samples (7 matched tumor samples before chemotherapy and at relapse)	n.m.	Array-based methylation profiling; pyrosequencing	CpG sites of 9/13 genes (*ARHGDIB*, *ARMCX2*, *COL1A*, *FLNA*, *FLNC*, *MEST*, *MLH1*, *NTS*, and *PSMB9*) acquired methylation in relapsed ovarian tumors after chemotherapy with CDDP.	[105]
*ASS1*	CDDP	OC	Frozen tumor tissue samples	54 patients (treated with surgery followed by post-operative CDDP chemotherapy, tissue sample at initial diagnosis and relapse)	n.m.	MSP	In a group of patients with methylated *ASS1* promoter at diagnosis, there were significantly more cases with partial clinical response, RFS < 12 months, or progressive disease; 34 patients relapsed during the study and, in 53% of them, methylation was present at diagnosis and in 74% at relapse. *ASS1* methylation at diagnosis was associated with significantly reduced RFS.	[106]
*FBXO32*	CDDP	OC	Tumor tissue surgical samples	96 OC patient samples and 5 normal benign gynecological disease cells	Median age 52 y.o. (18 to 90 y.o.)	COBRA; MSP; real-time qMSP	High *FBXO32* methylation level was significantly associated with higher stage and shorter PFS. Samples with higher *FBXO32* methylation disclosed lower expression.	[108]
*MAL*	CDDP	OC	Frozen tumor tissue samples	61 serous epithelial OC patient samples (60 III/IV stage, 26 living > 7 years, and 34 living < 3 years post-diagnosis; and 1 I/II stage cancer)	n.m.	Quantitative bisulfite sequencing; bisulfite sequencing; pyrosequencing; MSP	There was an average increase in *MAL* expression in III-IV stage ovarian tumors and transcript levels of short-term survivors compared to long-term survivors treated with CDDP. This was associated with CDDP resistance.	[128]
*SFRP5*	CDDP	OC	Frozen primary tumor biopsies	105 patient samples	n.m.	MSP; bisulfite sequencing	*SFRP5* methylation status was found to correlate with CDDP resistance in OC patients. The patients with no *SFRP5* methylation had a significantly better response to chemotherapy.	[113]
*SLFN11*	CDDP, carboplatin	OC, NSCLC	Tumor tissue samples	63 patient samples (41 in OC cohort and 22 in NSCLC cohort)	OC cohort: 5 ♀ < 50 y.o., 21 ♀ > 5 0 y.o., 15 ♀ unknown; NSCLC cohort: 10 ♂, 12 ♀ (2 < 50 y.o., 20 > 50 y.o.)	Infinium^®^ HumanMethylation450K BeadChip; bisulfite sequencing	*SLFN11* hypermethylation was associated with shorter OS and PFS. Clinical results paralleled those of cancer cell lines.	[114]
*PAX9*	CDDP	OC	Fresh frozen and FFPE tumor tissue samples	189 patient samples (129 FFPE and 57 frozen samples)	n.m.	Bisulfite sequencing; MSP; qMSP; whole-genome bisulfite sequencing; Infinium^®^ HumanMethylation450K BeadChip	Kaplan–Meier analysis showed that resistant/*PAX9*-methylated patients had reduced OS compared to cases without methylation. Moreover, patients with low *PAX9* expression disclosed shorter OS and recurrent disease.	[109]

Abbreviations: BC, breast cancer; BLCA, bladder cancer; CNS, central nervous system; COBRA, combined bisulfite restriction analysis; dCRT, definitive chemoradiotherapy; EOC, epithelial ovarian cancer; ESCC, esophageal squamous cell carcinoma; HBL, hepatoblastoma; HGSOC, high-grade serous ovarian carcinoma; HNSCC, head and neck squamous cell carcinoma; MALDI-TOF, matrix-assisted laser desorption/ionization-time of flight mass spectrometry; Me-DIP-seq, methylated DNA immunoprecipitation sequencing; MIBC, muscle-invasive bladder cancer; n.m., not mentioned; NPC, nasopharyngeal carcinoma; OC, ovarian cancer; OOSCC, oral and oropharyngeal squamous cell carcinoma; OS, overall survival; PC, prostate cancer; qMSP, quantitative methylation-specific PCR; RCC, renal cell cancer; RFS, relapse-free survival; RLGS, restriction landmark genomic scanning; RRBS, reduced representation bisulfite sequencing; SEOC, serous epithelial ovarian cancer; TCGA, The Cancer Genome Atlas; TNBC, triple-negative breast cancer; UCB, urothelial carcinoma; y.o., years old.

## Data Availability

All data analyzed during the current review are available in the PubMed repository, https://pubmed.ncbi.nlm.nih.gov (accessed on 1 February 2022).
